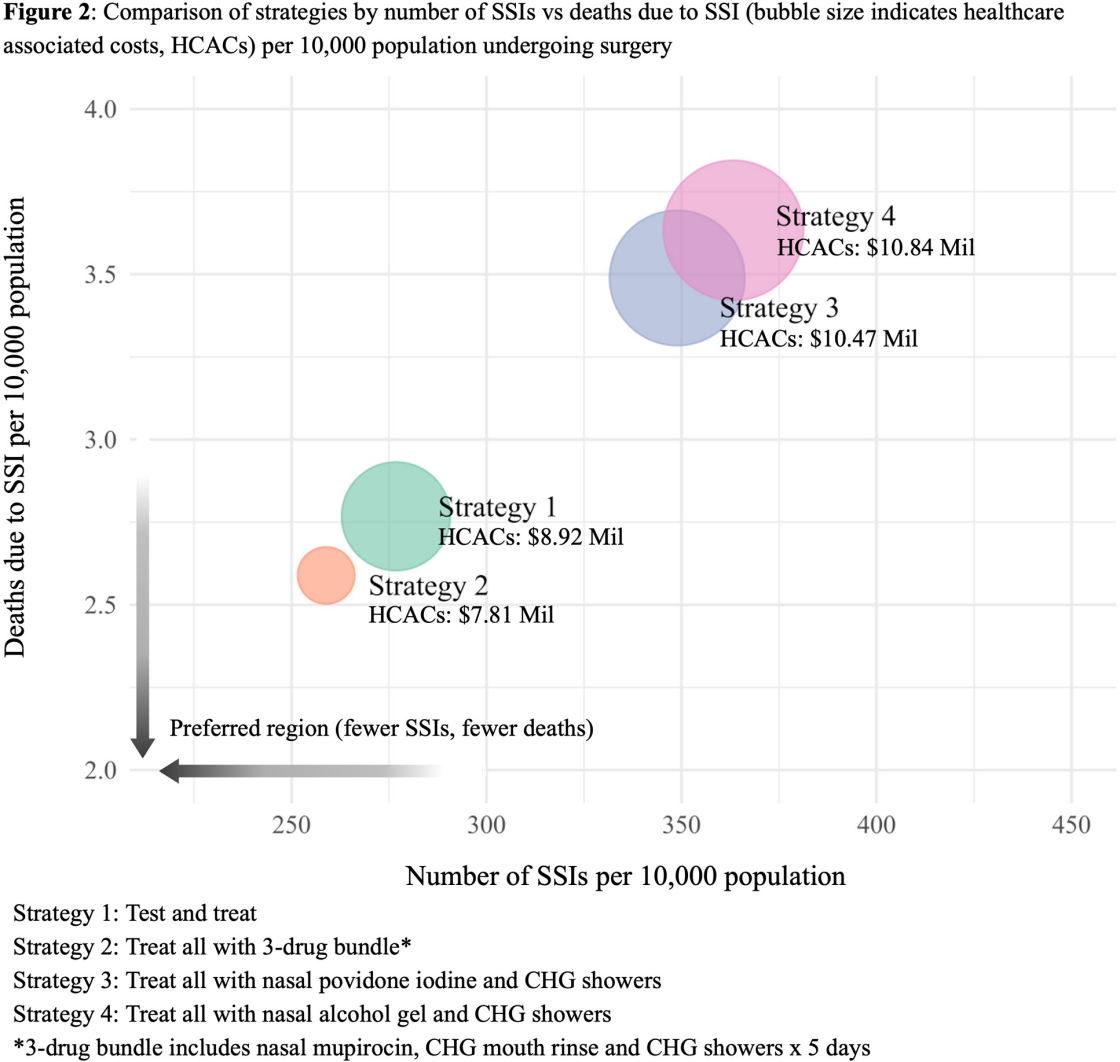# 50 Missing Pieces? SAAR Coverage and Its Role in Facility-Wide Antimicrobial Use Interpretation

**DOI:** 10.1017/ash.2026.10486

**Published:** 2026-06-23

**Authors:** Jon (Wei Yong) Ong, Shalini Kulasingam, Patricia Ferrieri, Susan Kline

**Affiliations:** 1 University of Minnesota

## Abstract

**Background:** Staphylococcus aureus (SA) colonization affects about 30% of U.S. adults and increases surgical-site infection risk 2 - 10 fold. A prior study by Kline et al. (2018) laid the foundation for evaluating efficacy and cost effectiveness of pre-operative decolonization interventions. This study evaluates an expanded set of up-to-date interventions, including: Strategy 1 - Individuals screening positive for SA receive a 5-day, 3-drug bundle. Non-SA carriers receive 2 pre-operative chlorhexidine gluconate (CHG) showers. Strategy 2 - All individuals receive the 3-drug bundle for 5 days. Strategy 3 - All individuals receive day-of-surgery nasal povidone iodine plus 2 CHG showers. Strategy 4 - All individuals receive day-of-surgery nasal alcohol gel plus 2 CHG showers. The 3-drug bundle consists of daily CHG showers, twice-daily CHG mouth rinse, and twice-daily nasal mupirocin ointment for 5 days leading up to surgery. **Methods:** A decision analytic model (Figure 1) simulated a U.S. adult population undergoing elective surgery over a 150-day horizon to evaluate the impact of the four strategies on surgical-site infections, SSI-related deaths, and healthcare-associated costs. Model parameters (Table 1) were derived from Kline et al. (2018), supplemental literature, and expert opinion. The model was used to determine SSI cases and costs per 10,000 population. One-way and probabilistic sensitivity analyses were conducted to determine the impact of uncertainty on choice of strategy. **Results:** Results are summarized in Table 2 and Figure 2. Strategy 2: Treat-all with 3-drug bundle, had the lowest healthcare-associated costs and prevented the most SSI cases, followed successively by Strategies 1, 3 and 4. These findings were robust across a series of one-way sensitivity analysis; the model was most sensitive to SSI probability and the relative risk associated with S. aureus colonization, while treatment efficacy parameters demonstrated limited impact on the overall results. In probabilistic sensitivity analyses, in which multiple parameters were varied, Strategy 2 remained the optimal strategy, whereas Strategy 4 was identified as the most expensive and least effective strategy. **Conclusion:** The results of this cost effectiveness analysis, combined with the results of our recent survey showing decolonization strategies vary nationally, highlight an opportunity to improve decolonization practices and outcomes.

**Figure:**
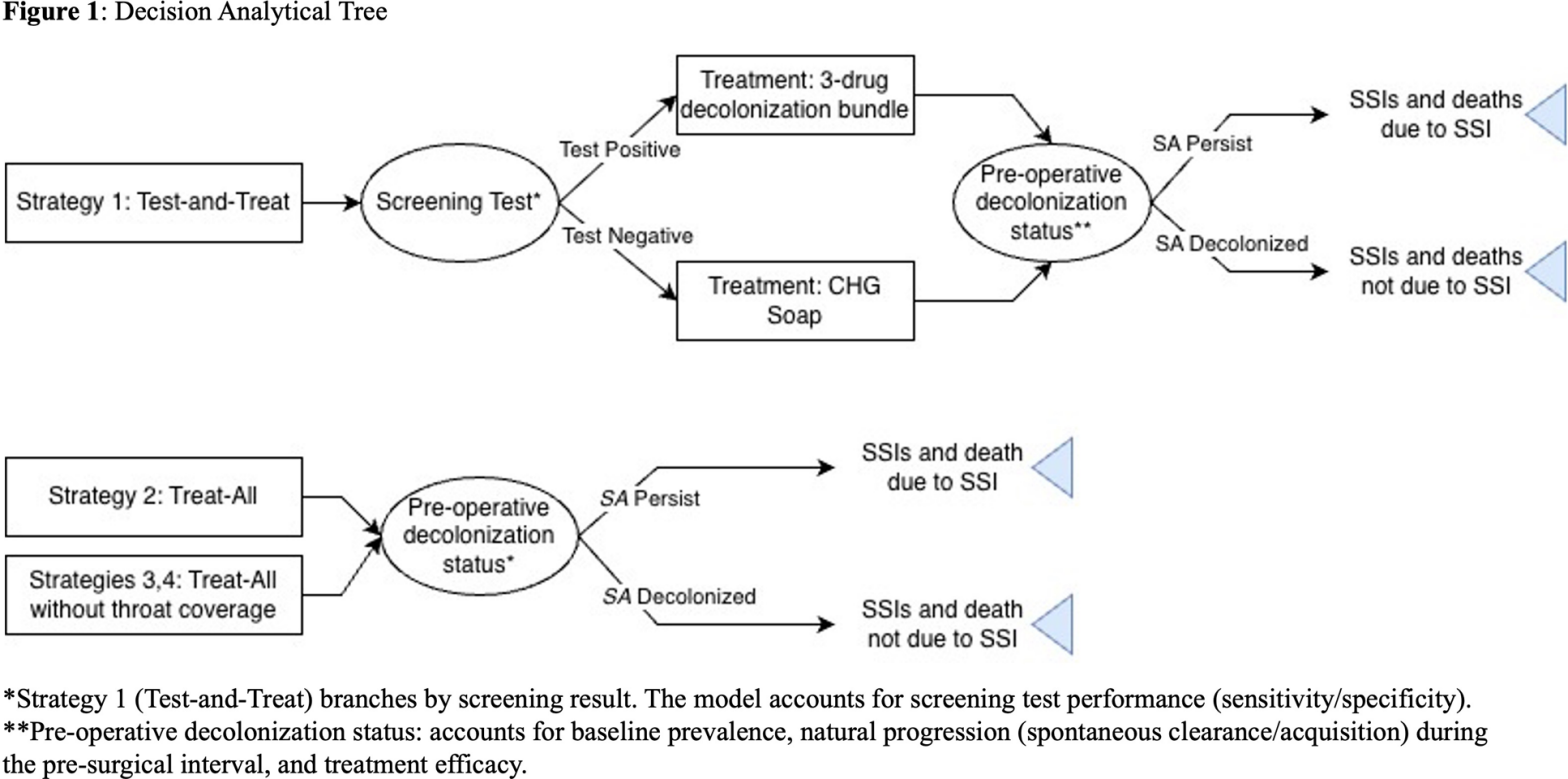

**Figure:**
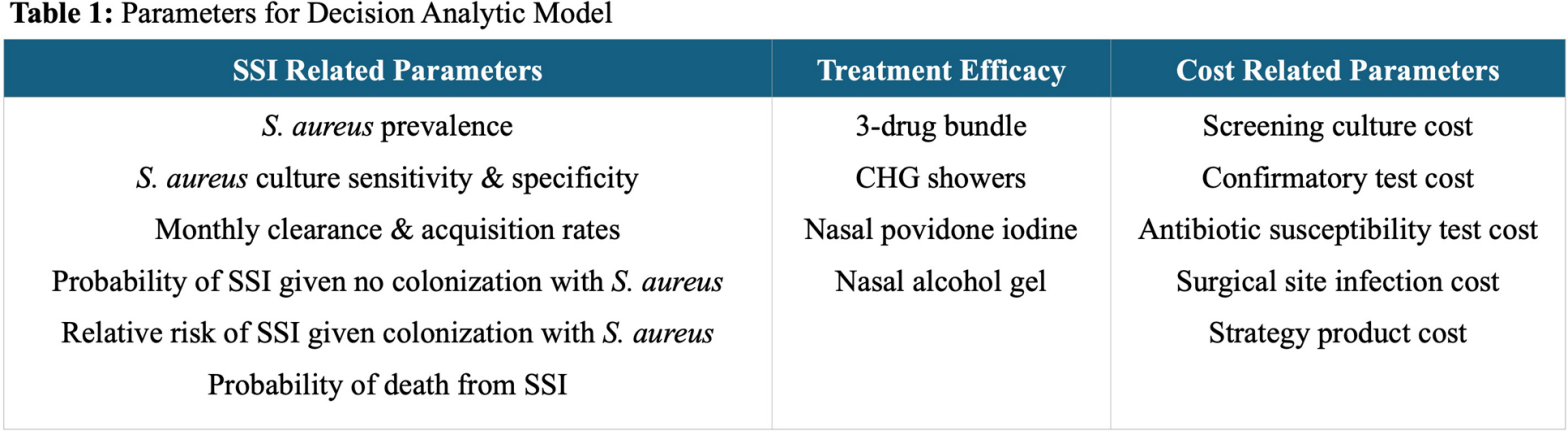

**Figure:**
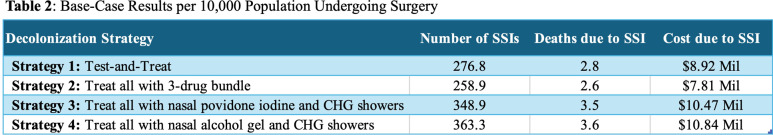

**Figure:**